# Spin-orbit torques associated with ferrimagnetic order in Pt/GdFeCo/MgO layers

**DOI:** 10.1038/s41598-018-24480-2

**Published:** 2018-04-16

**Authors:** JongHyuk Kim, DongJoon Lee, Kyung-Jin Lee, Byeong-Kwon Ju, Hyun Cheol Koo, Byoung-Chul Min, OukJae Lee

**Affiliations:** 1 Center for Spintronics, Korea Institute of Science and Technology, Seoul, 02792 Korea; 2 Department of Electrical Engineering, Korea University, Seoul, 02841 Korea; 3 KU-KIST Graduate School of Converging Science and Technology, Korea University, Seoul, 02841 Korea; 4 Department of Materials Science and Engineering, Korea University, Seoul, 02841 Korea; 5 Division of Nano and Information Technology, KIST school, Korea University of Science and Technology, Seoul, Korea

## Abstract

We investigate spin orbit torque (SOT) efficiencies and magnetic properties of Pt/GdFeCo/MgO multilayers by varying the thicknesses of GdFeCo and MgO layers. Our studies indicate that the ferrimagnetism in the GdFeCo alloy is considerably influenced by both thicknesses due to the diffusion of Gd atoms toward the MgO layer. Comparing to conventional Pt/ferromagnet/MgO structures, the Pt/GdFeCo/MgO exhibits a lower efficiency of SOTs associated with ferrimagnetic order and a similar magnitude of magnetic damping. The previous models that have been developed for rigid ferromagnets are inappropriate to analyze our experimental data, leading to an unphysical consequence of spin transmission larger than unity. Our results imply that the heavy-metal/ferrimagnet system is quite different from heavy-metal/ferromagnet systems in terms of magnetic dynamical modes, spin angular momentum transfer, and relaxation processes.

## Introduction

Significant amounts of works have demonstrated that spin-orbit torque (SOT) induced by in-plane current and strong spin-orbit coupling (SOC) can efficiently reorient the magnetization in heavy-metal/ferromagnet/oxide multilayers^1,2^. The origin of SOT in such hetero-structures is still under debate in spite of a number of intensive studies^3^. One of the most likely mechanisms that account for SOT is the spin-Hall effect (SHE)^1–4^ in the heavy-metal (HM); electrons in a longitudinal current are deflected transversely by spin-dependent scattering from defects or by Berry curvature of the band structure. The sign of transverse deflection depends on the sign of electron spin. This spin current is injected into the adjacent ferromagnet (FM), exerting spin torques on the magnetization. The other candidate is the Rashba-Edelstein effect^1–3,5^ (REE) that can generate a non-equilibrium spin polarization in conduction electron at interfaces or surfaces, resulting from the broken inversion symmetry with a strong SOC. Furthermore recent works have suggested that a spin transparency^6,7^ at the HM/FM interface also plays a role in determining the strengths of SOTs or that an interfacial SHE^8^ may generate spin currents as well. The agreement so far is that, regardless of its origin, there exhibit damping-like SOT (*τ*_*DL*_) proportional to $\overrightarrow{m}\times \overrightarrow{\sigma }\times \overrightarrow{m}$ and field-like SOT (*τ*_*FL*_) proportional to $\overrightarrow{m}\times \overrightarrow{\sigma }$, where the spin-polarization ($\overrightarrow{\sigma }$) is the injected spin vector which is in-plane and transverse to the applied charge current. The efficiencies of SOT are in general characterized by the conversion ratio of charge-to-spin current densities corresponding to the damping-like (DL) and field-like (FL) SOT, i.e. *θ*_*DL*_ and *θ*_*FL*_, respectively.

Recently the quest for appropriate magnetic materials has been extended from ferromagnet (FM) to ferrimagnet (F)^9–14^ and anti-ferromagnet (AFM)^15,16^, because the latter’s are beneficial for no stray field, low-power consumption, and fast magnetic manipulation. Amorphous GdFeCo alloy^17^, used in this paper, is one of the renowned rare-earth (RE) – transition metal (TM) ferrimagnetic^18,19^ materials. In the alloy, RE Gd 4*f* magnetic moments and TM FeCo 3*d* magnetic moments have “indirect” negative exchange interaction. This anti-parallel coupling arises from the bridge role of Gd 5*d* magnetic moments which are parallel to Gd 4*f* moments through direct exchange but anti-parallel to FeCo 3*d* magnetic moments via 5*d*-3*d* hybridization. The difference between Gd and FeCo magnetic moments results in small but finite net magnetization, making its magnetic state measurable. It is also well-known that the magnetic properties of GdFeCo can be tuned by temperature or its composition, and that the amorphous GdFeCo exhibits to have a bulk perpendicular magnetic anisotropy (PMA).

We are interested in how the ferrimagnetic order in GdFeCo is related to the SOT efficiency, spin transparency, and magnetic damping. Here, we report our investigations of SOT efficiencies and magnetic properties of Pt/GdFeCo/MgO multilayers using spin-torque ferromagnetic resonance (ST-FMR) technique. Our studies show that both the MgO and GdFeCo thicknesses considerably influence ferrimagnetism in the GdFeCo alloy so as to affect the magnitudes of *θ*_*DL*_ and *θ*_*FL*_. The measured SOT efficiencies of Pt/GdFeCo/MgO structures are smaller than those in conventional ferromagnetic layers, but the magnetic damping of the GdFeCo layer is similar to the magnitude of that in a conventional Pt/FM/MgO layer. We have examined whether the previous models that have been developed for rigid ferromagnets can explain our experimental results with GdFeCo ferrimagnets.

**Figure 1:**
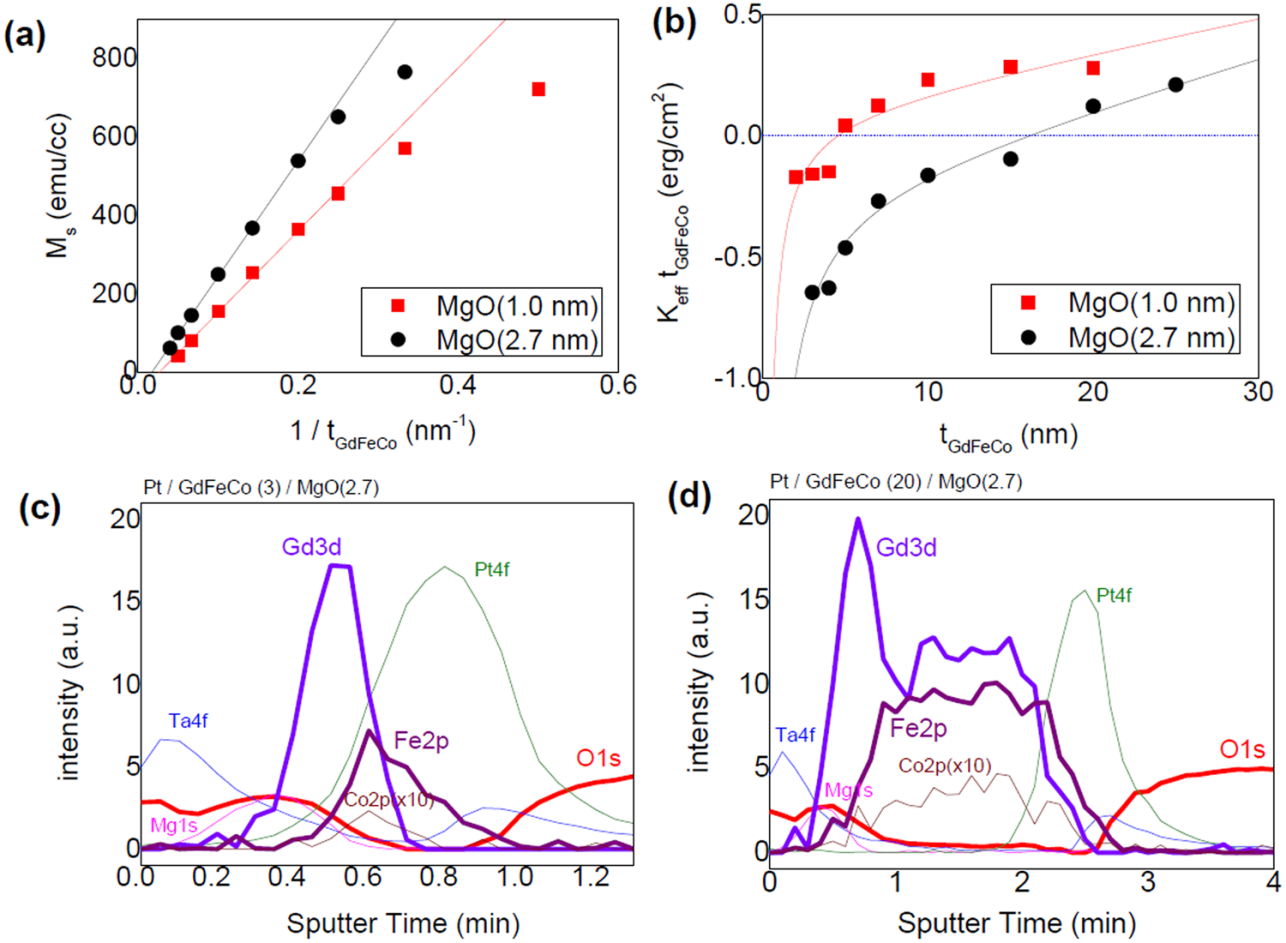
(**a**) Saturated net magnetization (*M*_*S*_) for un-patterned Pt/GdFeCo/MgO films at room temperature. The magnitude of *M*_*S*_ increases quasi-linearly with 1/*t*_*GdFeCo*_. (**b**) Effective anisotropy energy density $({K}_{eff}{t}_{GdFeCo})$ as a function of *t*_*GdFeCo*_. Solid lines are the fit to ${K}_{eff}{t}_{GdFeCo}=({K}_{V}-2\pi {M}_{S}^{2})\,{t}_{GdFeCo}+{K}_{S}$ with empirical ${M}_{s}\approx a+b/{t}_{GdFeCo}$. (**c**,**d**) Depth-profile of film composition for *t*_*GdFeCo*_ = 3 or 20 nm and *t*_*MgO*_ = 2.7 nm, using X-ray photoelectron spectroscopy (XPS). The results show a stronger peak of Gd 3*d* near the interface with the MgO layer.

The multilayers consist of Ta(1)/Pt(5)/Gd_25_Fe_65.6_Co_9.4_(*t*_*GdFeCo*_)/MgO(*t*_*MgO*_)/Ta(2) (thickness in nm) where *t*_*GdFeCo*_ was varied from 2 to 30 nm and *t*_*MgO*_ was 1.0 or 2.7 nm (see Methods for the film growth). The magnetic compensation temperature (*T*_*MC*_) of thick Gd_25_Fe_65.6_Co_9.4_ is around RT, and thus its angular momentum compensation temperature (*T*_*AC*_) is expected to be about 50–100 K higher than the *T*_*MC*_. A vibrating sample magnetometer (VSM) is used to measure the net magnetization (*M*_*s*_ = *M*_*FeCo*_ − *M*_*Gd*_) and the effective out-of-plane anisotropy field $({H}_{k}^{eff}=2{K}_{eff}/{M}_{s})$ of the un-patterned Pt/GdFeCo/MgO films at room temperature (RT). A peculiar thickness dependence was observed in the magnetization measurements. Figure 1(a) shows that *M*_*s*_ of the GdFeCo is proportional to 1/*t*_*GdFeCo*_ in a quasi-linear manner; when the GdFeCo thickness is the same, the film stack with a thicker MgO layer shows a higher *M*_*s*_.

To further understand this peculiarity, we investigated the depth profile of film composition using X-ray photoelectron spectroscopy (XPS). Figure 1(c,d) illustrate the composition-depth profile for the films with *t*_*GdFeCo*_ = 3 or 20 nm and *t*_*MgO*_ = 2.7 nm. The stronger peaks of Gd 3*d* adjacent to the MgO layer in both samples suggest a diffusion of Gd atoms toward MgO and a possible formation of a thin GdO_x_ layer on top of the alloy. These are energetically favorable processes, since the enthalpy of formation of Gd_2_O_3_ (≈−1800 kJ/mol) is much lower than that of MgO (≈−600 kJ/mol). Therefore FeCo is richer in the vicinity of the Pt layer whereas Gd and GdO_x_ are richer in the proximity to MgO. The formation of GdO leads to the loss of Gd moments, and, as a consequence, gives rise to a peculiar thickness dependence of magnetization. The degree of ferrimagnetic order, i.e. the amount of anti-parallel magnetic coupling between Gd and FeCo moments, is inversely proportional to net magnetization *M*_*s*_; a small *M*_*s*_ implies that the ferrimagnetic order is recovered with thicker GdFeCo and thinner MgO layers.

Magnetic anisotropy of Pt/GdFeCo/MgO also has interesting GdFeCo and MgO thickness dependences. Figure 1(b) summarizes the thickness dependence of the measured effective anisotropy energy density, ${K}_{eff}{t}_{GdFeCo}$ vs *t*_*GdFeCo*_, showing that the multilayers preferentially exhibit a larger PMA (i.e. *K*_*eff*_ > 0) with a larger *t*_*GdFeCo*_ and with a smaller *t*_*MgO*_. The former is definitely due to a strong bulk anisotropy energy density (*K*_*V*_) of GdFeCo, and the latter is due to the weaker oxidation of Gd atoms. The enhancement of PMA is also consistent with the decrease of *M*_*s*_, i.e. the recovery of the ferrimagnetic order in the amorphous GdFeCo layer. The effective anisotropy energy density is usually expressed as ${K}_{eff}{t}_{GdFeCo}=({K}_{V}-2\pi {M}_{S}^{2})\,{t}_{GdFeCo}+{K}_{S}$, that can be transformed to ${K}_{eff}{t}_{GdFeCo}\approx ({K}_{V}-2\pi {a}^{2})\,{t}_{GdFeCo}-2\pi {b}^{2}/{t}_{GdFeCo}+({K}_{S}-4\pi ab)$ with the empirical relation of ${M}_{s}\approx a+b/{t}_{GdFeCo}$. Here *K*_*S*_ is an interface anisotropy energy density. By fitting the data in Fig. 1(a,b) to the equations, we obtain ${K}_{v}\approx 2.1\times {10}^{5}\,erg/c{m}^{3}$ and ${K}_{S}\approx -\,0.4\,erg/c{m}^{2}$ for *t*_*MgO*_ = 2.7 nm and ${K}_{v}\approx 1.6\times {10}^{5}\,erg/c{m}^{3}$ and ${K}_{S}\approx -\,0.05\,erg/c{m}^{2}$ for *t*_*MgO*_ = 1.0 nm on the assumption that both *K*_*v*_ and *K*_*S*_ remain constant *w.r.t. t*_*GdFeCo*_.

Next step is to investigate the resistance and the effective anisotropic magnetoresistance (AMR) of Pt/GdFeCo/MgO films as a function of *t*_*GdFeCo*_. In order to estimate the SOT efficiencies, *θ*_*DL*_ and *θ*_*FL*_, in the ST-FMR measurement, it is necessary to obtain both the resistance, ${R}_{o}({t}_{GdFeCo})$, and the effective AMR, ${\rm{\Delta }}{R}_{AMR}^{eff}({t}_{GdFeCo})$, for each ST-FMR^20^ device. We measured $R(\phi )={R}_{o}+{\rm{\Delta }}{R}_{AMR}^{eff}\,co{s}^{2}\,({\rm{\phi }}-{{\rm{\phi }}}_{o})$ under a rotating magnetic field within the sample plane (see Methods for the device fabrication). Here φ is the angle between the FeCo magnetization and the current and φ_*o*_ is the offset angle. We note that ${\rm{\Delta }}{R}_{AMR}^{eff}$ is contributed from both ordinary AMR and spin-Hall magneto-resistance^21^ (SMR). The device without the GdFeCo layer (i.e. Ta/Pt/MgO/Ta layer) has a resistance ${R}_{o}({t}_{GdFeCo}=0)$ of 298 Ω and thus the resistivity of Pt $({\rho }_{Pt(5)})$ on top of Ta (1) buffer layer is 45 μΩ cm.

**Figure 2:**
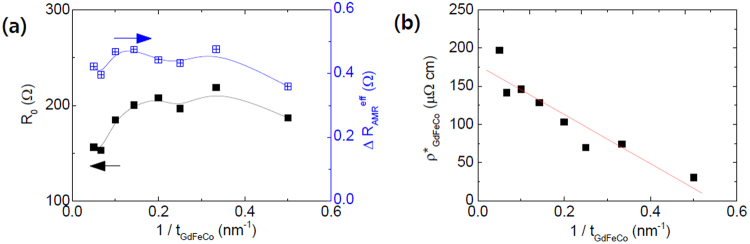
(**a**) Device resistance (*R*_*o*_) and effective AMR $({\rm{\Delta }}{R}_{AMR}^{eff})$ as a function of *t*_*GdFeCo*_ for *t*_*MgO*_ = 2.7 nm. The device resistance without the GdFeCo layer was ${R}_{o}({t}_{GFC}=0)\approx 298\,{\rm{\Omega }}$ and thus ${\rho }_{Pt(5)}\approx 45\,{\rm{\mu }}{\rm{\Omega }}\cdot {\rm{cm}}$. (**b**) Resistivity of GdFeCo layer $({{\rho }^{\ast }}_{GdFeCo})$ obtained by the parallel resistance model.

Based on the measured ${R}_{o}({t}_{GdFeCo})$ in Fig. 2a, we estimated the resistivity of the GdFeCo layer $({{\rho }^{\ast }}_{GdFeCo})$, using the expression of ${{\rho }^{\ast }}_{GdFeCo}\,({t}_{GdFeCo})=\frac{w}{l}{t}_{GdFeCo}{(\frac{1}{R({t}_{GdFeCo})}-\frac{1}{{R}_{o}(0)})}^{-1}$ within the parallel resistance model, where *w* is a channel width and *l* is a channel length. As illustrated in Fig. 2b, the ${{\rho }^{\ast }}_{GdFeCo}\,({t}_{GdFeCo})$ *unexpectedly* increases with increasing *t*_*GdFeCo*_ and the linear fit to the thickness-dependent resistivity with Fuchs-Sondheimer’s model^22^, ${{\rho }^{\ast }}_{GdFeCo}\,({t}_{GdFeCo})={\rho }_{b}+\frac{{\rho }_{s}}{{t}_{GdFeCo}}$, provides the bulk contribution ${\rho }_{b}=178\,{\rm{\mu }}{\rm{\Omega }}\cdot \mathrm{cm}\,$and the surface contribution ${\rho }_{s}=-323\,{\rm{\mu }}{\rm{\Omega }}\cdot {\rm{cm}}\cdot {\rm{nm}}$. Certainly the *negative* value of *ρ*_*s*_ is uncommon, and it is unlikely that less surface scattering occurs with a thinner metallic GdFeCo layer. Instead, we have to take into account the influence of antiferromagnetic order on the resistivity; as *t*_*GdFeCo*_ increases, the GdFeCo layer smoothly is transformed from a non-compensated ferromagnetic-like material to a compensated anti-ferromagnetic-like material. A stronger spin-dependent scattering in anti-ferromagnetic order results in higher resistivity than those with ferromagnetic order^23,24^.

**Figure 3:**
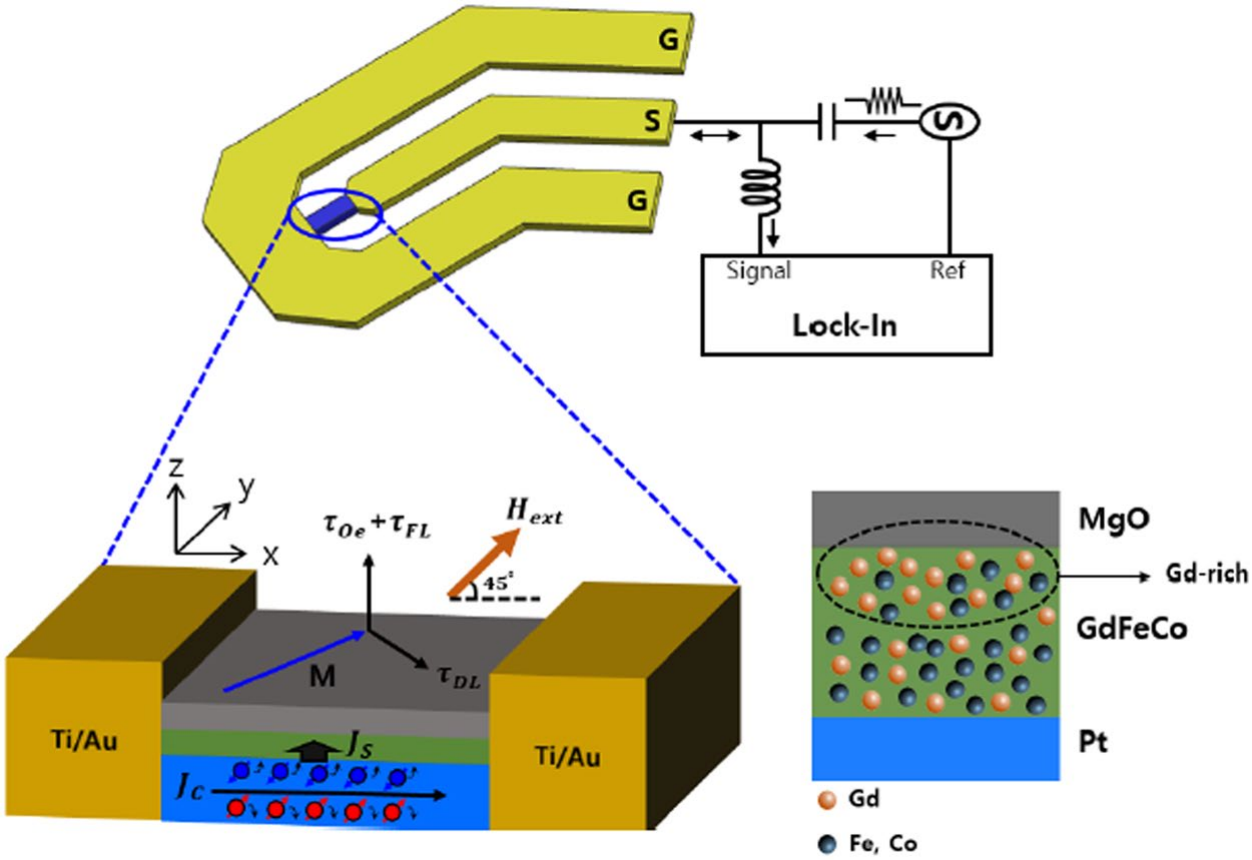
Schematic of ST-FMR measurement. Inserted diagram illustrates the diffusion of Gd atoms toward MgO and a possible formation of a thin GdO_x_ layer at the top of the alloy. The FeCo is richer in the vicinity of the Pt layer whereas Gd and GdO_x_ richer in proximity to MgO.

**Figure 4:**
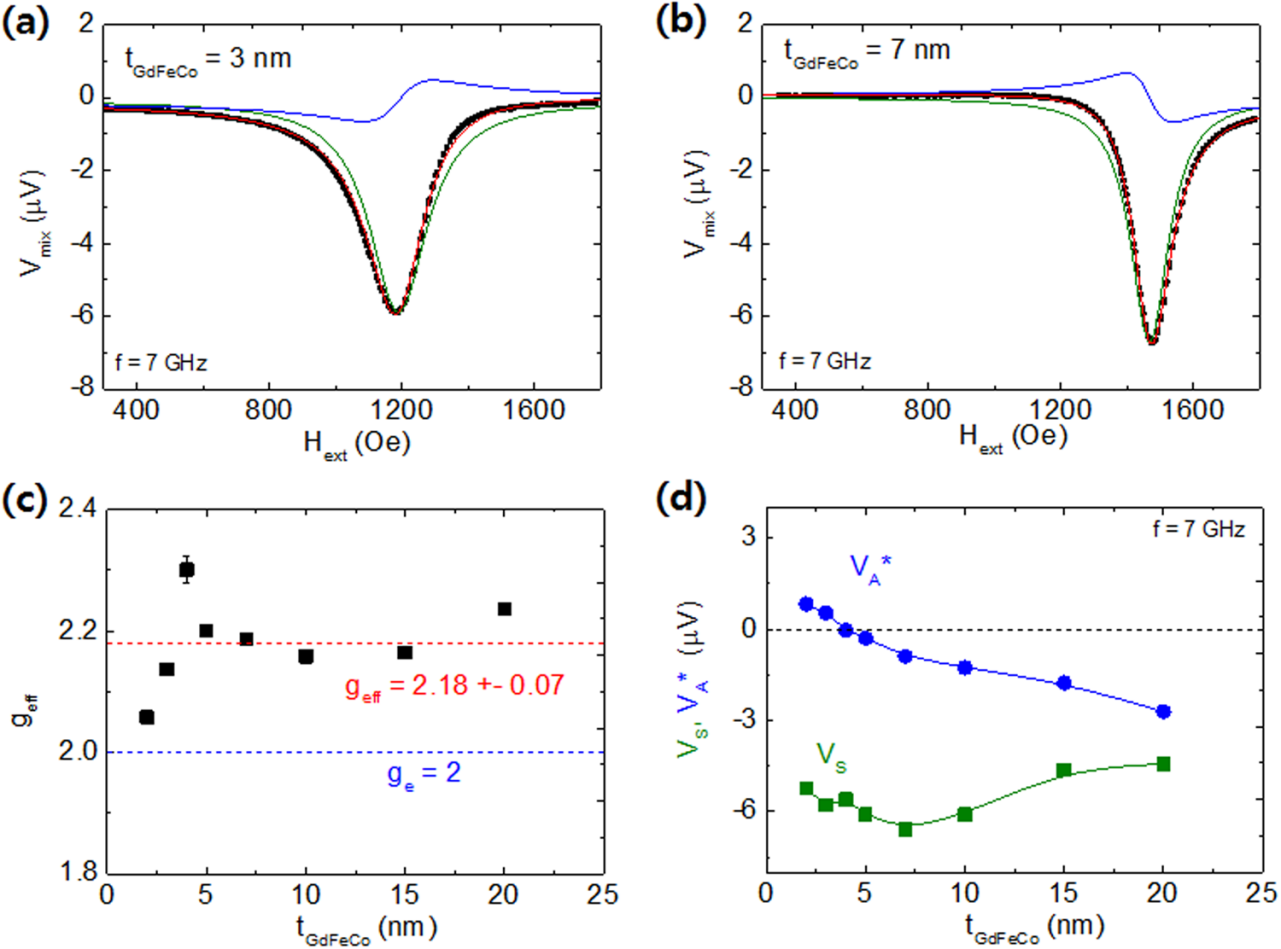
(**a**,**b**) Representative spectra of ST-FMR for the devices with *t*_*GdFeCo*_ = 3 and 7 nm at 7 GHz. All of the measured curves are very well fit to the Eq. (1) (red curve). The symmetric (green) and anti-symmetric (blue) parts of the signals are also plotted. (**c**) Obtained effective g-factor, *g*_*eff*_, of the GdFeCo layer. The averaged *g*_*eff*_ from all devices is 2.18 ± 0.07. (**d**) Obtained *V*_*S*_ and ${V}_{A}^{\ast }\equiv {V}_{A}\,{(1+4\pi {M}_{eff}/{H}_{res})}^{-1/2}$ that are directly proportional to the DLT (= DL-SOT) and the FLT (= OFT + FL-SOT) respectively.

We systematically measured ST-FMR spectra to examine the SOT efficiency and the magnetic damping of Pt/GdFeCo(*t*_*GdFeCo*_)/MgO(2.7 nm) samples as a function of *t*_*GdFeCo*_ (see Fig. 3 and Methods for ST-FMR measurement). The measured voltage, *V*_*mix*_(*H*), is in general a sum of symmetric and anti-symmetric Lorentzians given by,

1 $${V}_{mix}(H)={V}_{o}+[{V}_{S}\frac{{\Delta }{H}^{2}}{{(H-{H}_{res})}^{2}+{({\Delta }H)}^{2}}+{V}_{A}\frac{(H-{H}_{res}){\Delta }H}{{(H-{H}_{res})}^{2}+{\Delta }{H}^{2}}]$$

where *V*_*o*_ is a background voltage (mostly negligible), *V*_*S*_ (*V*_*A*_) is a symmetric (anti-symmetric) term of resonance amplitude, *ΔH* is a half width at half maximum, and *H*_*res*_ is a ferromagnetic resonance field. Figure 4(a,b) show representative spectra for the devices with *t*_*GdFeCo*_ = 3 nm and 7 nm at 7 GHz. All of the measured curves are very well fit to the Eq. (1) (red curve) and from the fit we obtained *H*_*res*_, *ΔH*, *V*_*S*_, and *V*_*A*_ at different frequencies for each sample. The measured ST-FMR voltages were decomposed to the symmetric (green) and anti-symmetric (blue) parts of the signals.

From the theory of magnetic resonance^25,26^, a dynamical system containing two magnetic sublattices has two normal modes of oscillation: in-phase and out-of-phase motions. The in-phase motion between Gd and FeCo moments corresponds to an ordinary FMR whose oscillation frequency is determined by Kittel’s theory, but with an effective gyromagnetic ratio (γ_*eff*_) instead of electron’s gyromagnetic ratio (γ_*e*_). The ${{\rm{\gamma }}}_{eff}=-\,\frac{{g}_{eff}\,{\mu }_{B}}{\hslash }$, where *μ*_*B*_ is Bohr magneton and *g*_*eff*_ is an effective g-factor (associated with SOC strength in a material). The net angular momentum of electrons in RE-TM alloy is expressed as $({M}_{FeCo}-{M}_{Gd})/{{\rm{\gamma }}}_{eff}={M}_{FeCo}/{{\rm{\gamma }}}_{FeCo}-{M}_{Gd}/{{\rm{\gamma }}}_{Gd}$, where ${M}_{FeCo}({M}_{Gd})$ is the magnetization from the FeCo (Gd) subnetwork and ${{\rm{\gamma }}}_{FeCo}\,({{\rm{\gamma }}}_{Gd})$ is the corresponding gyromagnetic ratio. The γ_*eff*_ is determined as the ratio of the net magnetization to the net angular momentum of the system, i.e. ${{\rm{\gamma }}}_{eff}=\frac{({M}_{FeCo}-{M}_{Gd}\,)}{({M}_{FeCo}/{{\rm{\gamma }}}_{FeCo}-{M}_{Gd}/{{\rm{\gamma }}}_{Gd})}$. On the other hand, the exchange field between Gd and FeCo moments dominates the other normal mode, the out-of-phase precession. The mode frequency is in general very high, which is one or two orders of magnitude higher than the in-phase mode. In our ST-FMR measurements, the applied frequency range corresponds to the regular FMR mode, thus we interpret our results based on the analysis from the usual macrospin model/theory with the experimental γ_*eff*_. We note that this interpretation with the experimental γ_*eff*_ is not valid in general and is significantly violated especially when the ferrimagnetic system is close to the angular momentum compensation condition^27^.

Here we determined the magnitudes of *γ*_*eff*_ and 4*πM*_*eff*_ (effective demagnetization field) from the frequency (*f*) dependence of resonant peak position (*H*_*res*_) using Kittel’s equation for a magnetic film with an in-plane magnetic field: ${(2\pi f)}^{2}={({\gamma }_{eff})}^{2}{H}_{res}({H}_{res}+4\pi {M}_{eff})$. The *g*_*eff*_ of the GdFeCo layer is expected to be in the range of g-factors^28–30^ of individual materials, Gd, Fe and Co; ${g}_{Gd}\approx 2.0$, ${g}_{Fe}\approx 2.1$, and *g*_*Co*_ ≈ 2.15. Indeed, the averaged *g*_*eff*_ from all devices is consistently within the range of 2.18 ± 0.07, as shown in Fig. 4(c), but exhibiting no clear thickness dependence. We note that other RE-TM alloys^28,29^, e.g. Tb-FeCo or Dy-FeCo, may have quite different values of *g*_*eff*_ deviated from those of the subnetwork RE materials; *g*_*Tb*_ ≈ 1.5 and *g*_*Dy*_ ≈ 1.2.

The ST-FMR theory^7^ implies that the symmetric part of FMR (*V*_*S*_) is proportional to DL-SOT (*τ*_*DL*_) acting on the GdFeCo magnetization while the anti-symmetric part (*V*_*A*_) originates from the sum of FL-SOT (*τ*_*FL*_) and Oersted field torque (OFT, *τ*_*Oe*_);

2 $${V}_{S}\propto {\gamma }_{eff}{\tau }_{DL}={\gamma }_{eff}\frac{\hslash }{2e}\frac{{J}_{Pt}}{4\pi {M}_{s}{t}_{GFC}}{\theta }_{DL}$$

3 $${V}_{A}\propto {\gamma }_{eff}({\tau }_{Oe}+{\tau }_{FL})\sqrt{1+\frac{4\pi {M}_{eff}}{{H}_{res}}}\approx {\gamma }_{eff}(\frac{{t}_{Pt}{J}_{Pt}}{2}+\frac{\hslash }{2e}\frac{{J}_{Pt}}{4\pi {M}_{s}{t}_{GFC}}{\theta }_{FL}\,)\sqrt{1+\frac{4\pi {M}_{eff}}{{H}_{res}}}$$

where *ħ* is Planck’s constant, *e* is the electron charge, *J*_*Pt*_ is the current density through the underlying Pt, *t*_*Pt*_ is the thickness of Pt (=5 nm in this paper). The measured voltages are interpreted to magnetic precession angles of less than 0.5°, corresponding to a linear-response regime. Thus we ignored the contribution in *Vs* from spin-pumping & inverse SHE (SP-ISHE), which are estimated to be less than 0.2 μV. Figure 4(d) summarizes the obtained *V*_*S*_ and ${V}_{A}^{\ast }\equiv {V}_{A}\,{(1+4\pi {M}_{eff}/{H}_{res})}^{-1/2}$ that are directly proportional to the damping-like torque (DLT) and nominal field-like torque (FLT = FL-SOT + OFT) respectively. The sign of ${V}_{A}^{\ast }$ changes at ${t}_{GdFeCo}\approx 5\,nm$, indicating the presence of a FL-SOT that is comparable but opposite to the OFT. The sign of *V*_*S*_ is the same as that of conventional Pt/FM bilayers, i.e. *θ*_*DL*_ > 0.

**Figure 5:**
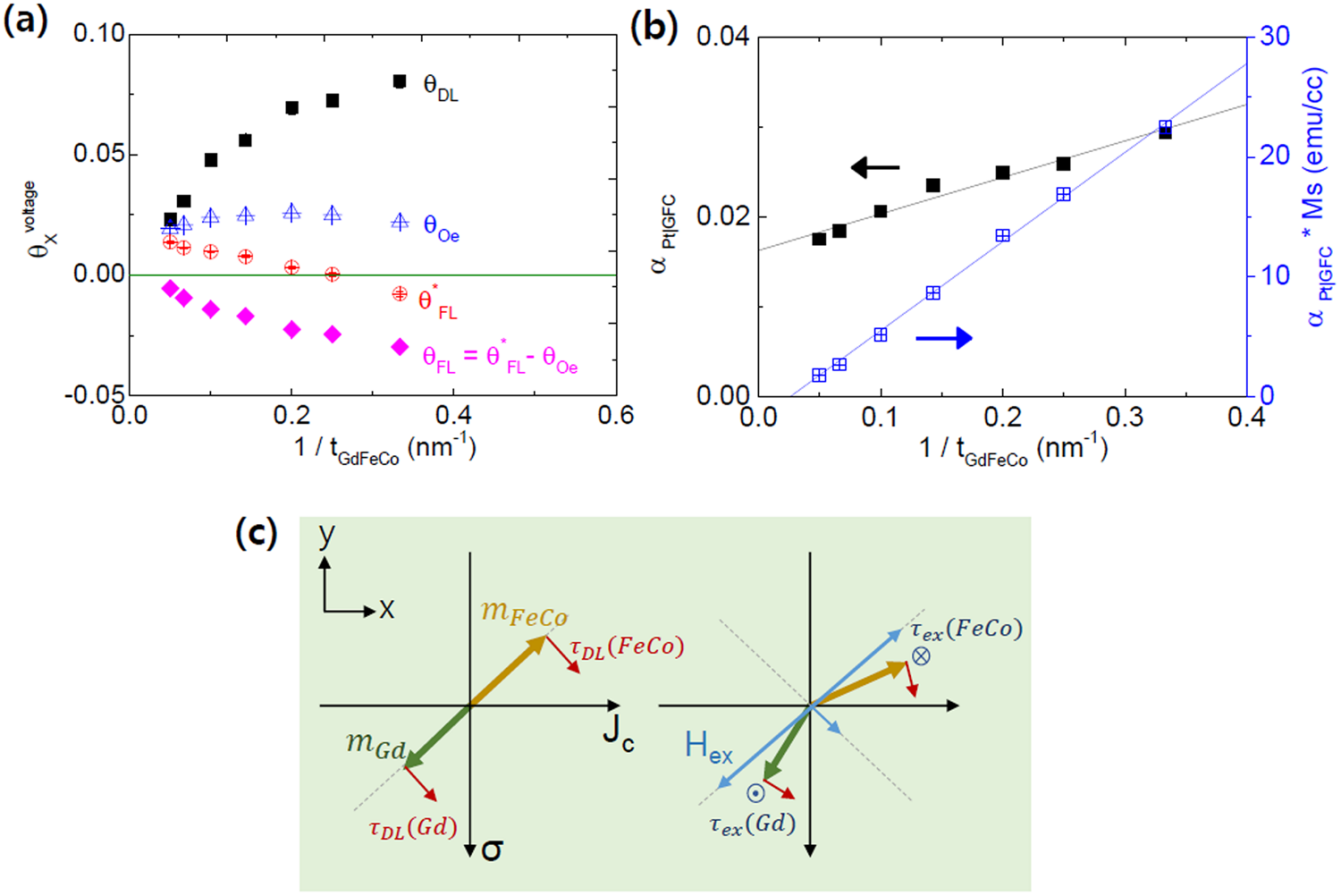
(**a**) Estimated SOT efficiencies (*θ*_*DL*_, *θ*_*FL*_) as a function of *t*_*GdFeCo*_ as well as the measured ${\theta }_{FL}^{\ast }$ and calculated *θ*_*Oe*_. The values of *θ*_*DL*_ and ${\theta }_{FL}^{\ast }$ are obtained from the voltage amplitudes (*V*_*S*_ and ${V}_{A}^{\ast }$) using Eq. (4). (**b**) Experimental *α*_*eff*_ and *α*_*eff*_ *M*_*S*_ *w.r.t*. 1/*t*_*GdFeCo*_, determined from *ΔH*. (**c**) Rotational directions of DL-SOT onto the FeCo and Gd moments are opposite for a given spin-polarization (σ). Then the staggered magnetization induces exchange-fields that can develop a higher dynamical mode in the range of a few hundreds of GHz to THz.

We determined *θ*_*DL*_ from the voltage amplitude of ST-FMR^31,32^ signal (*V*_*S*_) using the expression given by

4 $${V}_{S}=-\frac{{\gamma }_{eff}}{4\,}[\frac{sin\phi \,co{s}^{2}\phi }{{\rm{\Delta }}H\,2\pi \,{(df/dH)}_{{H}_{res}}}]\,[\frac{\hslash }{2e}\frac{1}{{M}_{s}{t}_{GdFeCo}}]\,[\frac{{R}_{dev}}{{R}_{o}w{t}_{Pt}}]\,{\rm{\Delta }}{R}_{AMR}^{eff}\,{I}_{rf}^{2}\,{\theta }_{DL}$$

where φ = π/4 in our measurements. The quantitative magnitude of nominal ${\theta }_{FL}^{\ast }$ from the ${V}_{A}^{\ast }$ was obtained in the same manner. Here the fraction of current flowing through the underlying Pt layer was considered with the parallel resistance model. The RF current through a device (*I*_*rf*_) was estimated for a given RF power and frequency using a vector network analyzer (VNA) and taking into account the loss factors. We also obtained the effective FL-SOT efficiency (*θ*_*FL*_), what we are interested in, by using ${\theta }_{Oe}=\frac{e}{\hslash }4\pi {M}_{S}{t}_{Pt}{t}_{GdFeCo}$ and ${\theta }_{FL}={\theta }_{FL}^{\ast }-{\theta }_{Oe}$. Figure 5(a) summarizes the experimental *θ*_*DL*_ and ${\theta }_{FL}^{\ast }$ as well as the calculated *θ*_*Oe*_ and *θ*_*FL*_. The signs of *θ*_*DL*_ and *θ*_*FL*_ are opposite to each other, which is consistent with those of Pt/FM layers reported previously^1,7^. The current-induced FL-SOT appears adversely to the OFT. The most interesting feature of the results is that both *θ*_*DL*_ and *θ*_*FL*_ are strongly dependent on *t*_*GdFeCo*_ that maybe unusual for the conventional ferromagnets. This implies that the SOT in HM/FI may not arise entirely from the bulk spin Hall effect, but from other interfacial contributions. For thin GdFeCo layers, each *θ*_*DL*_ and *θ*_*FL*_ approach closely to 0.08 and −0.03 which are quite similar to ones in conventional Pt/FM bilayers. The FeCo-rich phase of GdFeCo as a consequence of the Gd oxidation would lead to the SOT efficiencies (*θ*_*DL*_ and *θ*_*FL*_) similar to conventional ferromagnets. The magnitude of *θ*_*DL*_ and *θ*_*FL*_ are inversely proportional to *t*_*GdFeCo*_ (Fig. 5a) and, the SOT efficiencies are strongly correlated with the degree of ferrimagnet order.

It is tempting to apply the interface spin-transparency model to explain the correlation between the SOT efficiencies and the degree of ferrimagnetic order. According to the recent spin-transparency model, an effective spin-mixing conductance at the HM/FM (or HM/FI) interface quantifies the transmission of spin current, and plays an important role in determining the effective magnitudes of *θ*_*DL*_ and *θ*_*FL*_. Based on our measured magnitudes, this model expects a relatively smaller spin-mixing conductance at the Pt|GdFeCo interface than conventional Pt|FM’s. We have examined if this is the case.

In order to estimate the effective spin-mixing conductance, the magnetic damping (*α*_*eff*_) was investigated as a function of *t*_*GdFeCo*_ from the linewidth (Δ*H*) of each FMR peak, using the relation, ${\rm{\Delta }}H={\rm{\Delta }}{H}_{0}+(2\pi {\alpha }_{eff}/\,{\gamma }_{eff})f$ where Δ*H*_0_ is an inhomogeneous linewidth broadening. Figure 5(b) shows the experimental *α*_*eff*_ and *α*_*eff*_*M*_*S*_ as a function of 1/*t*_*GdFeCo*_. The measured *α*_*eff*_ in Pt/GdFeCo/MgO was 0.015~0.03, which is similar to the *α*_*eff*_ in conventional Pt/TM-FM metals (e.g. Co, CoFeB or Py)/MgO samples^6,7^. According to Bailey *et al*.^33^, even small doping of RE (e.g. Dy, Tb, Ho) into TM-FM can strongly enhance magnetic damping at least over one order of magnitude, but it is not the case with Gd and Eu. The magnetic damping of RE-TM is qualitatively associated with the magnetic character of localized RE 4*f* shell. Most of RE atoms, e.g. Tb, Dy and Ho, possess an orbital angular moment (*L* ≠ 0) which can couple the spin moment (*S*) to the lattice. As a result, the magnetic relaxation to the lattice becomes very effective. By contrast, the half-filled Gd 4*f* shell has *S* = 7/2 but *L* = 0, consequently zero SOC (*g*_*Gd*_ ≈ 2.0), and therefore the magnetic damping with Gd is not significantly enhanced as we observed in our experiments.

The enhancement of damping in a FM/HM bilayer is often attributed to spin pumping^34^ (SP) or two-magnon scattering^35,36^. The spin mixing conductance is calculated from the data shown in Fig. 5b, where the *α*_*eff*_*M*_*S*_ linearly increases with *1/t*_*GdFeCo*_. If this thickness dependence were solely from SP, the Pt/GdFeCo interface would have the effective spin-mixing conductance, ${g}_{eff}^{\uparrow \downarrow }=\frac{4\pi }{\gamma \hslash }\frac{\partial ({M}_{s}\alpha )}{\partial (1/{t}_{GdFeCo})}\approx 52\,n{m}^{-2}$, and the spin-transparency, $T\approx \frac{2({e}^{2}/h){g}_{eff}^{\uparrow \downarrow }}{1/{\lambda }_{Pt}{\rho }_{Pt}}\approx 1.8$, where *λ*_*Pt*_ is the spin-diffusion length^7,20^ in Pt (≈1 *nm*). The assumption gives rise to a certainly un-physical consequence (*T* > 1), indicating that the assumption is not correct and there exists another contribution to the magnetic damping. The enhancement of *α*_*eff*_ could be due to the two-magnon scattering process possibly originating from the significant oxidation of Gd atoms at the interface, which becomes increasingly dominant especially with thin GdFeCo layers, as we have discussed earlier. Another accurate experiment and further theoretical works are required to explicitly separate the SP contribution from the damping enhancement in order to obtain correct spin-transparency and ${g}_{eff}^{\uparrow \downarrow }$ at the Pt/MgO interface.

The previous models that have been developed for rigid ferromagnets is inappropriate to analyze our experimental data, and it is necessary to have a caution in their applications to ferrimagnets. The presence of ferrimagnetic order gives rise to an unexpected contribution to the magnetic dynamics that is distinguished from the passive role of interface in the spin transparency model. The most distinct property of the ferrimagnet is that the dynamics in two magnetic sub-lattices are governed by the coupled equations of the net magnetization, $m({\rm{t}})={m}_{FeCo}(t)+{m}_{Gd}(t)$, and the Néel order parameter, $l({\rm{t}})={m}_{FeCo}(t)-{m}_{Gd}(t)$ as suggested by recent theoretical works^37–39^. The DL-SOT in ferrimagnet gives rise to a dynamical mode of *m*_*FeCo*_ and *m*_*Gd*_ oscillation at high frequencies (1–10 GHz). Figure 5(c) shows the rotational directions of DL-SOT onto the FeCo and Gd moments which are opposite to each other for a given spin-polarization (σ). Then the staggered magnetization induces exchange-fields that can develop a higher dynamical mode in the range of a few hundreds of GHz to THz. We note that this description is in analog with a recent theoretical expectation^39^ in which a current-induced DL-SOT can emit a THz spin-wave in an antiferromagnetic domain-wall motion. In such scenarios, the out-of-phase motion is rather important than a ferromagnetic in-phase mode and the DL-SOT might become seemingly less efficient in the ST-FMR experiment, as the anti-ferromagnetic order recovers with increasing *t*_*GdFeCo*_. In order to understand the magnetic dynamics and to extract material parameters correctly in the ferrimagnetic dynamics, it is required to develop more advanced models and deliberated mathematical works that describe delicate dynamics in ferrimagnets with both DL- and FL- SOTs. The THz dynamics of magnetization should be studied using an experimental apparatus that can support THz electrical signals.

In summary, we investigated the SOT efficiencies and magnetic properties of the Pt/GdFeCo/MgO multilayers with varying MgO and GdFeCo thicknesses using several measurement techniques including spin-torque ferromagnetic resonance (ST-FMR). Our work demonstrated that both the MgO and GdFeCo thicknesses considerably influence the ferrimagnetic order in the GdFeCo alloy so as to affect the magnitudes of *θ*_*DL*_ and *θ*_*FL*_. The SOT efficiencies are smaller than those in conventional Pt/FM/MgO layers. On the other hand the magnetic damping of the GdFeCo was similar to the latter’s. The previous models developed for ferromagnetic dynamics is not proper to describe the complex aspect of ferrimagnetic dynamics at the interface of HM/FI bilayers. Further theoretical/experimental works are required to understand the magnetic dynamics, spin current transmission^40^, and relaxation mechanism at the interface of HM/FI bilayers that is significantly different from those at the interface of HM/FM bilayers.

## Methods

### Thin film growth

The multilayer films were prepared on thermally oxidized Si substrates by DC/RF magnetron sputtering at room temperature (RT). The multilayer structures consist of, from the substrate side, Ta(1)/Pt(5)/Gd_25_Fe_65.6_Co_9.4_(*t*_*GdFeCo*_)/MgO(*t*_*MgO*_)/Ta(2) (nominal thickness in nm) where *t*_*GdFeCo*_ was varied from 2 to 30 nm and *t*_*MgO*_ was 1.0 or 2.7 nm. The GdFeCo alloy used in this paper has a magnetization compensation temperature (*T*_*MC*_) at around RT, at which the moments cancel each other and the alloy has no net magnetization. A 1-nm-thick Ta film was employed as a wetting layer, while the MgO/Ta capping layer was to protect its under-layers and expected to be fully air-oxidized. The base pressure of the chamber was <5 × 10^−8^ Torr, the deposition rates were low (<0.5 Å*/*s). No post annealing was carried out except during the fabrication because ferrimagnetism and/or perpendicular magnetic anisotropy (PMA) can be substantially damaged due to the crystallization of the GdFeCo layer after that.

### Device fabrication

For the ST-FMR measurement, we used optical lithography and Ar ion-milling to fabricate the multilayer films into rectangular shaped bars with 15 μm width (*w*) and 50 μm length (*l*). In a subsequent process step, a waveguide contact made of Ti (10 nm)/Au (100 nm) was defined on top of the samples so as to pass a RF current through the devices. We note that the maximum temperature was 110 °C during the fabrication. All the measurements were performed at RT and we focused on the films with *t*_*MgO*_ = 2.7 nm.

### ST-FMR measurement

The schematic of circuit diagram of ST-FMR measurement are illustrated in Fig. 3. A pulsed microwave signal in the range 3–8 GHz with a nominal output power of 10 dBm was applied to the samples having in-plane magnetic anisotropy (i.e. *K*_*eff*_  < 0). In the meantime, an external magnetic field (from −1.8 kOe to +1.8 kOe) was swept at an angle of 45° within the plane of the sample. The applied *I*_*rf*_ generates an oscillating transverse spin current through SHE or REE that is injected into the adjacent GdFeCo layer and that then exerts spin-torques on the magnetic moments of GdFeCo layer (via spin-angular momentum transfer to local FeCo 3*d* and Gd 4*f* moments). At around resonances, the magnetic precession in the GdFeCo layer is caused by oscillating SOTs, thereby producing an oscillating magnetoresistance of the Pt/GdFeCo layer at the frequency of *I*_*rf*_. The mixing of an oscillatory resistance and *I*_*rf*_ passing through the GdFeCo generates a finite dc voltage (*V*_*mix*_), which is simultaneously detected with a lock-in amplifier connected to the DC port of the bias tee.
